# Cardioprotective Effects of Transfusion of Late-Phase Preconditioned Plasma May Be Induced by Activating the Reperfusion Injury Salvage Kinase Pathway but Not the Survivor Activating Factor Enhancement Pathway in Rats

**DOI:** 10.1155/2017/8526561

**Published:** 2017-07-30

**Authors:** Yang Zhao, Zhi-nan Zheng, Yan-na Pi, Xue Liang, San-qing Jin

**Affiliations:** Department of Anesthesia, The Sixth Affiliated Hospital, Sun Yat-sen University, No. 26 Yuancunerheng Road, Guangzhou 510655, China

## Abstract

A previous study in our laboratory demonstrated that transfusion of plasma collected at the late phase of remote ischemic preconditioning (RIPC) could reduce myocardial infarct size. Here, we tested whether the reperfusion injury salvage kinase (RISK) and survivor activating factor enhancement (SAFE) pathways are involved in transferring protection. In a two-part study, donor rats (*n* = 3) donated plasma 48 hours after RIPC (preconditioned plasma) or control (nonpreconditioned plasma). Normal (part 1) or ischemic (part 2) myocardia were collected from recipients (*n* = 6) 24 hours after receiving normal saline, nonpreconditioned plasma, and preconditioned plasma or after further suffering ischemia reperfusion. Western blot was performed to analyze STAT3, Akt, and Erk1/2 phosphorylation in normal and ischemic myocardium (central area and border area). In normal myocardia, preconditioned plasma increased Akt and Erk1/2 phosphorylation significantly compared to nonpreconditioned plasma and normal saline; no STAT3 phosphorylation was detected. In ischemic myocardia, preconditioned plasma increased Akt and Erk1/2 phosphorylation significantly in both central and border areas compared to other fluids; no significant difference in STAT3 phosphorylation occurred among groups. Transfusion of preconditioned plasma collected at the late phase of RIPC could activate the RISK but not SAFE pathway, suggesting that RISK pathway may be involved in transferring protection.

## 1. Introduction

Remote ischemic preconditioning (RIPC) has been shown to be a safe and effective strategy to attenuate myocardial ischemia reperfusion (IR) injury [1, 2] and offers early-phase as well as late-phase protection [3, 4]. Previous studies have found that the cardioprotective effects induced by RIPC at the early phase can be transferred between individuals [5–7]. Our studies found that late-phase protection of RIPC could also be transferred by plasma between individuals and that transfusion of late-phase preconditioned plasma can improve blood pressure recovery [8] and reduce infarct size after myocardial IR [9]. However, the signal transduction mechanism underlying the transferred protection of late-phase preconditioned plasma remains unclear.

The reperfusion injury salvage kinase (RISK) pathway, a term given to describe a group of survival protein kinases that includes Akt and extracelluar signal-regulated kinases 1/2 (Erk1/2), confers powerful cardioprotection and represents a novel target thereof [10]. Hausenloy et al. found that phosphorylation of Akt and Erk1/2 was essential to mediate IPC-induced protection [11, 12]. Heidbreder et al. showed that the activation of Erk1/2 was involved in the early-phase protection induced by RIPC, and Cai et al. demonstrated that the activation of Akt via the IL-10 receptor mediated the late-phase protection induced by RIPC [13, 14]. Additionally, the survivor activating factor enhancement (SAFE) pathway, which involves Janus kinase (JAK) and signal transducer and activator of transcription 3 (STAT-3) signaling, has also been shown to be involved in the early-phase and late-phase cardioprotection induced by IPC [15, 16].

However, whether the transference of late-phase protection of RIPC shares established cardioprotective signaling pathways, such as the RISK or SAFE pathway, remains to be clarified. Therefore, using an *in vivo* myocardial IR rat model, the aim of this study was to test whether the preconditioned plasma collected at the late phase of RIPC from donor rats could activate the RISK and/or SAFE pathway in the recipient myocardium.

## 2. Methods

**Figure 1:**
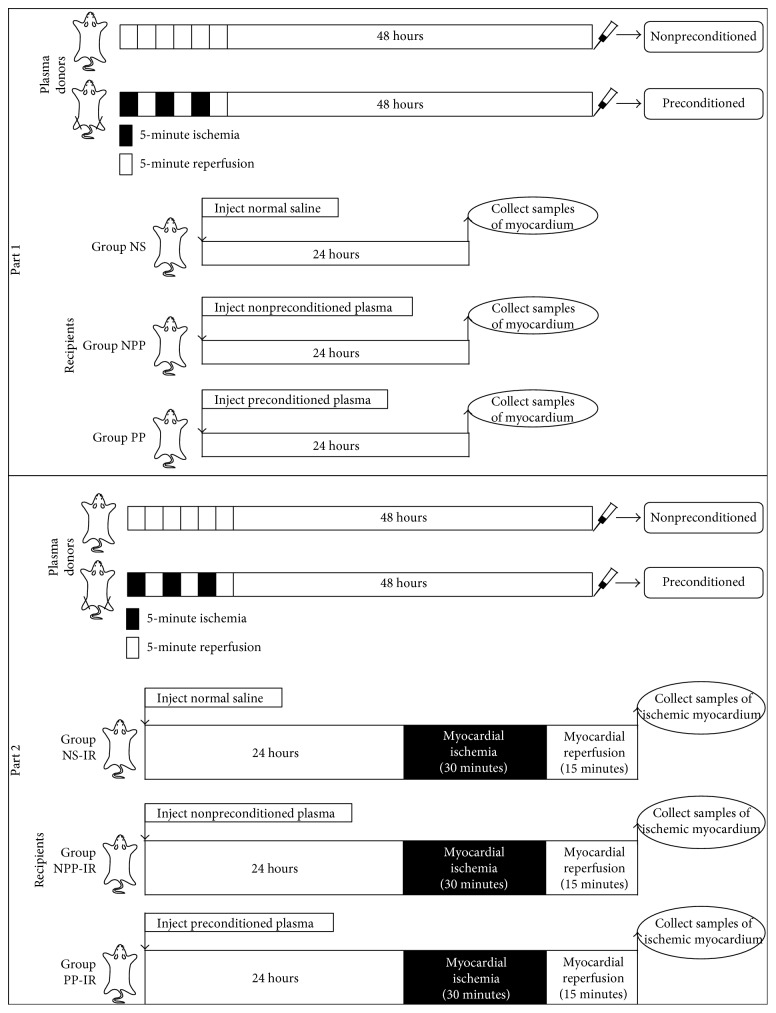
Experimental protocols. The experiment was divided into two parts. Part 1 was designed to ascertain the impact of preconditioned plasma on survival kinase phosphorylation in normal myocardium that was not subjected to myocardial ischemia reperfusion, and part 2 was designed to ascertain the impact in ischemic myocardium, including the central area of the area at risk and the border area of the area at risk, which was obtained after myocardial ischemia reperfusion. After samples of myocardium were collected, Western blots were performed on these samples to detect the phosphorylation of survival kinases.

The experiment was divided into two parts (Figure 1). Part 1 was designed to ascertain the impact of preconditioned plasma on survival kinase phosphorylation in normal myocardium that was not subjected to myocardial IR, and part 2 was designed to ascertain the impact in IR myocardium, including the central area of the area at risk and the border area of the area at risk, which was obtained after myocardial IR.

### 2.1. Ethics

All animal protocols were approved by the Institutional Animal Care and Use Committee of Sun Yat-sen University (LAEC-2012-0602).

### 2.2. Part 1: Phosphorylation of Survival Kinases in the Myocardia after Preconditioned Plasma Transfusion prior to Ischemia

#### 2.2.1. Animals and Grouping

We randomized 24 male Lewis rats (Vital River Company, Beijing, China; 10–12 weeks old) into 5 groups: 2 groups of plasma donor rats (*n* = 3) and 3 groups of study rats (*n* = 6). Plasma donor rats were further randomized depending on whether transient limb ischemia was induced to the rat or not (*n* = 3 for each group). Study rats received fluid transfusion but did not suffer myocardial IR and were randomized to 3 groups (*n* = 6 for each group) depending on the type of fluid transfused: normal saline (group NS), nonpreconditioned plasma (group NPP), or preconditioned plasma (group PP).

#### 2.2.2. Transient Limb Ischemia [8, 9]

Intraperitoneal pentobarbital (50 mg/kg) was administered to plasma donor rats for anesthesia. Then, the rats were subjected (or not) to transient limb ischemia. Transient limb ischemia was induced by binding elastic rubber bands around both proximal hind limbs for 5 minutes, followed by 5 minutes of reperfusion by releasing the noninvasive ligature. Cessation of blood flow to the hind limbs was confirmed by using a laser Doppler to monitor hind limb microcirculatory blood flow (Laser Doppler Blood Perfusion Imager, Perimed AB, Järfälla, Sweden). Elastic bands were placed on both hind limbs of plasma donor rats that did not suffer transient limb ischemia but were not tied for 40 minutes.

#### 2.2.3. Plasma Preparation and Transfusion [8, 9]

At 48 hours after finishing the whole preconditioning or control protocol, 8–10 ml blood was drawn through the abdominal aorta into anticoagulant tubes after the rats were reanesthetized by intraperitoneal pentobarbital (50 mg/kg). All plasma donor rats were euthanized through hypovolemia-induced cardiac arrest under anesthesia after the blood samples were collected. The whole blood was centrifuged at 4°C and 1690*g* for 10 minutes to prepare the plasma. Preconditioned plasma was a resultant from the rats undergoing ischemia preconditioning, whereas nonpreconditioned plasma was obtained from the rats not subjected to transient limb ischemia. According to the grouping, 2 ml of normal saline or 2 ml of preconditioned plasma or 2 ml of nonpreconditioned plasma was transfused into the assigned study rats immediately following plasma preparation through the caudal vein at a rate of 1 ml/min.

#### 2.2.4. Preparation of Myocardium Samples and Immunoblotting of Survival Kinases

At 24 hours after fluid transfusion, all study rats were anesthetized with intraperitoneal pentobarbital (60 mg/kg) and then their hearts were quickly removed. The left ventricular tissue was excised and frozen in liquid nitrogen, then promptly transferred and stored at −80°C. Western blots were performed on the myocardium from the left ventricle. Frozen myocardium samples precooled to the temperature of liquid nitrogen were powdered in a mortar and pestle. Powdered ventricular tissue (100 mg) weighed by an electronic analytic balance (KERN, Balingen, Germany) was used for protein extraction. Frozen myocardium samples were homogenized on ice with 1 ml ice-cold RIPA lysis buffer (50 mM Tris, pH 7.5, 150 mM NaCl, 1% Nonidet-P40, 1% deoxycholic acid, and 0.1% sodium dodecyl sulfate) (Sigma, St. Louis, MO, USA), 125 *μ*l phosphatase inhibitor cocktail (Roche Applied Science, Mannheim, Germany), and 125 *μ*l protease inhibitor cocktail (Complete mini, Roche Applied Science, Mannheim, Germany). The homogenate was centrifuged at 12,400*g* at 4°C for 30 minutes, and the resulting supernatant was collected. Protein concentration was determined using a Pierce BCA protein assay kit (Pierce, Rockford, IL, USA) according to the manufacturer's instructions. Equal amounts of total protein (40 *μ*g/sample) were heated to 95°C for 10 minutes in sample loading buffer (Beyotime, Shanghai, China) and then loaded on 10% polyacrylamide gels with 5% stacking gels. The gels underwent electrophoresis at 60 V for 30 minutes and 100 V for 2 hours (25 mM Tris, 192 mM glycine) (Sigma). The proteins in the gels were then transferred to polyvinylidene difluoride membranes (Millipore, Billerica, MA, USA) at 100 V for 100 minutes in ice-cold transferring buffer (25 mM Tris, 192 mM glycine, 20% (*v*/*v*) methanol) (Sigma).

The membranes were blocked in Tris-buffered saline (170 mM NaCl, 50 mM Tris, pH 7.4) with 0.1% Tween (TBS-T) (Sigma) containing 5% nonfat milk for 1 hour at 25°C. The membranes were then washed in TBS-T three times (15 minutes each time) and incubated for 12 hours at 4°C with antibodies against ^705^Tyr-phospho-STAT3 (CST-9145), ^473^Ser-phospho-Akt (CST-4060), ^202^Thr/^204^Tyr-phospho-Erk1/2 (CST-4370), and GAPDH (CST-3683) (1 : 1000, Cell Signaling Technology, Danvers, MA, USA). Membranes were then washed three times (15 minutes each time) in TBS-T followed by incubation with anti-rabbit IgG, HRP-linked antibody (CST-7074, USA) for 1 hour at 26°C, and then washed three times in TBS-T (15 minutes each time). Protein bands were visualized by enhanced chemiluminescence and then the membranes were exposed to X-ray films (Kodak, USA). The membranes were then stripped in stripping buffer (Beyotime) and reprobed with antibodies against total STAT3 (CST-4904), Akt (CST-4691), and Erk1/2 (CST-4695) (1 : 1000, Cell Signaling Technology). GAPDH was used to ensure equal protein loading. Films were scanned into digital images. Densitometric analysis was performed using Quantity One 4·62 (Bio-Rad, Hercules, CA, USA). Phosphorylation of STAT3, Akt, and Erk1/2 was expressed as the ratio of phosphorylated protein level to total protein level.

### 2.3. Part 2: Phosphorylation of Survival Kinases in the IR-Exposed Myocardium at 24 Hours following Preconditioned Plasma Transfusion

#### 2.3.1. Animals and Grouping

We randomized 24 male Lewis rats (Vital River Company, 10–12 weeks old) into 5 groups: 2 groups of plasma donor rats (transient limb ischemia was induced or not; *n* = 3 for each group) and 3 groups of IR rats (*n* = 6 for each group). IR rats underwent myocardial IR 24 hours after fluid transfusion. They were randomized to group NS-IR (received normal saline), group NPP-IR (received nonpreconditioned plasma), or group PP-IR (received preconditioned plasma).

#### 2.3.2. Transient Limb Ischemia

The procedures of transient limb ischemia were the same as described in Section 2.2.2.

#### 2.3.3. Plasma Preparation and Transfusion

The procedures of plasma preparation and transfusion were the same as described in Section 2.2.3.

#### 2.3.4. Myocardial IR Procedures [8, 9]

The myocardial IR model was established as described in our previous studies [8, 9]. Briefly, IR rats were anesthetized with intraperitoneal pentobarbital (60 mg/kg), intubated, and ventilated (Harvard Rodent Ventilator, Harvard Apparatus, Holliston, MA, USA) with room air, a tidal volume of 8–10 ml/kg, and a respiratory rate of 70–80/min. The right jugular vein was cannulated for fluid administration, and the right carotid artery was cannulated for blood pressure monitoring (Harvard transducer). Electrocardiogram was monitored continuously using a BIOPAC system (BIOPAC Systems, Goleta, CA, USA) throughout the experiment. The rectal temperature was maintained at 36.8–37.2°C by placing the rat on a heating pad (Nuanfeng Heating Element Company, Foshan, China) and carefully adjusting the level of heating. Left thoracotomy was performed between the third and fourth ribs, and then the left anterior descending coronary artery was identified and ligated with a 7–0 polypropylene suture tunneled under the left anterior descending coronary artery. A slipknot was tied over a section of cotton thread placed directly over the vessel to create the occlusion. Occlusion was deemed successful when the myocardium supplied by the vessel turned pale. After 30 minutes of ischemia, the slipknot was released by gently pulling the slipknot sutures in opposite directions and then the cotton thread was pulled out. At this time, reperfusion began and lasted for 15 minutes.

#### 2.3.5. Preparation of Heart Samples and Immunoblotting of Survival Kinases

At the end of the 15-minute reperfusion, the polypropylene suture tunneled under the left anterior descending coronary artery was retied and 4 ml of 2% Evans blue (Sigma) was administered intravenously to distinguish the area at risk (unstained portion of the myocardium) from the area not at risk (Evans blue-stained portion of the myocardium). The heart was excised and placed on ice, and then the right ventricle was removed. The unstained portion of the myocardium was carefully removed and divided into the border area (the area adjacent to the Evans blue-stained portion) and the central area. Western blots were performed on myocardia from the border and central areas. The procedures for Western blotting were same as those described in Section 2.2.4.

### 2.4. Statistical Analysis

The data were analyzed using SPSS 16.0 (SPSS Inc., Chicago, IL, USA). All values are expressed as the means ± standard deviation of the mean (SD). One-way analysis of variance (ANOVA) with the least significant difference tests for multiple comparisons was used to analyze the effects of fluid type on survival kinase phosphorylation. Differences were regarded as statistically significant when *P* < 0.05.

## 3. Results

### 3.1. Phosphorylation of Survival Kinases in Normal Myocardium Obtained 24 Hours following Fluid Transfusion (Figure 2)

Preconditioned plasma transfusion resulted in significant increases in Akt phosphorylation compared to nonpreconditioned plasma transfusion or normal saline transfusion (group PP 2.27 ± 0.26 versus group NPP 1.50 ± 0.58, *P* = 0.024, and group PP 2.27 ± 0.26 versus group NS 1.52 ± 0.6, *P* = 0.026). However, there was no significant difference in Akt phosphorylation between groups NPP and NS (*P* > 0.05).

Preconditioned plasma transfusion resulted in significant increases in Erk1/2 phosphorylation compared to nonpreconditioned plasma transfusion and normal saline transfusion (group PP 2.07 ± 0.96 versus group NPP 1.05 ± 0.35, *P* = 0.013, and group PP 2.07 ± 0.96 versus group NS 0.72 ± 0.36, *P* = 0.002). However, there was no significant difference in Erk1/2 phosphorylation between groups NPP and NS (*P* > 0.05).

Phosphorylation of STAT3 was not observed in group NS, NPP, or PP.

### 3.2. Phosphorylation of Survival Kinases in IR Myocardium Obtained after Myocardial IR 24 Hours after Fluid Transfusion

#### 3.2.1. Phosphorylation of Survival Kinases in the Central Area of the Area at Risk (Figure 3)

The central area of the area at risk was collected at 15 minutes after myocardial reperfusion. There was a significant increase of Akt phosphorylation in the central area of the area at risk in group PP-IR compared to that in either group NPP-IR or group NS-IR (group PP-IR 2.26 ± 0.35 versus group NPP-IR 1.60 ± 0.65, *P* = 0.025, and group PP-IR 2.26 ± 0.35 versus group NS-IR, 1.06 ± 0.32, *P* = 0.000). However, Akt phosphorylation in group NPP-IR did not differ from that in group NS-IR (*P* > 0.05).

There was a significant increase of Erk1/2 phosphorylation in the central area of the area at risk in group PP-IR compared to that in group NPP-IR or group NS-IR (group PP-IR 2.00 ± 0.32 versus group NPP-IR 1.41 ± 0.33, *P* = 0.017, and group PP-IR 2.00 ± 0.32 versus group NS-IR 1.25 ± 0.46, *P* = 0.004), whereas phosphorylation of Erk1/2 in group NPP-IR did not differ from that in group NS-IR (*P* > 0.05).

There was no significant difference in STAT3 phosphorylation in the central area of the area at risk among group PP-IR, NPP-IR, or NS-IR (group PP-IR 1.28 ± 0.65, group NPP-IR 1.53 ± 0.55, and group NS-IR 1.04 ± 0.64; *P* > 0.05).

#### 3.2.2. Phosphorylation of Survival Kinases in the Border Area of the Area at Risk (Figure 4)

The border area of the area at risk was collected at 15 minutes after myocardial reperfusion. Akt phosphorylation in the border area of the area at risk in group PP-IR was significantly increased compared to that in groups NPP-IR and NS-IR (group PP-IR 2.14 ± 0.23 versus group NPP-IR 1.59 ± 0.35, *P* = 0.028, and group PP-IR 2.14 ± 0.23 versus group NS-IR 1.46 ± 0.53, *P* = 0.009). However, Akt phosphorylation in group NPP-IR did not differ from that in group NS-IR (*P* > 0.05).

Erk1/2 phosphorylation in the border area of the area at risk in group PP-IR was significantly increased compared to that in groups NPP-IR and NS-IR (group PP-IR 2.72 ± 0.68 versus group NPP-IR 1.54 ± 0.58, *P* = 0.007, and group PP-IR 2.72 ± 0.68 versus group NS-IR 1.75 ± 0.67, *P* = 0.020). Erk1/2 phosphorylation in group NPP-IR did not differ from that in group NS-IR (*P* > 0.05).

There was no significant difference in STAT3 phosphorylation in the border area of the area at risk among group PP-IR, NPP-IR, and NS-IR (group PP-IR 2.78 ± 1.22, group NPP-IR 2.05 ± 0.66, and group NS-IR 1.69 ± 0.81; *P* > 0.05).

## 4. Discussion

At 24 hours after transfusion, preconditioned plasma collected at the late phase of RIPC could activate the RISK pathway but not the SAFE pathway in recipient myocardium both prior to and following myocardial IR, suggesting that the RISK pathway rather than the SAFE pathway may be involved in the signal transduction for transferring late-phase protection of RIPC by plasma.

Previous studies have shown that RISK pathway activation may represent an important mechanism of the cardioprotection induced by IPC and RIPC [11–14, 17–19]. However, previous studies demonstrating the involvement of the RISK pathway in transferred protection of RIPC are limited. Breivik et al. [20] found that coronary effluent collected from one isolated rat heart model after IPC treatment offered cytoprotection via PI3K/Akt-dependent signaling upon reperfusion in another. The RISK pathway consists of both PI3K/Akt and Erk1/2 pathways, but Breivik et al. [20] only confirmed the important role of the PI3K/Akt pathway in the myocardial protection induced by preconditioned coronary effluent and did not research the role of the Erk1/2 pathway. In contrast, our study demonstrated that the preconditioned plasma increased both Akt phosphorylation and Erk1/2 phosphorylation in recipient myocardium. Hausenloy et al. [12] found that IPC induced a biphasic response in Akt and Erk1/2 phosphorylation in isolated perfused rat hearts, indicating that IPC resulted in an immediate increase in Akt and Erk1/2 phosphorylation, which declined during the ischemia period followed by a second increase at reperfusion. However, the study by Heidbreder et al. [13] showed that the RIPC induced by occlusion of the superior mesenteric artery increased Erk1/2 phosphorylation in the small intestine whereas it did not alter Erk1/2 phosphorylation in the myocardium. In comparison, our results showed that the transfusion of preconditioned plasma could activate the RISK pathway in the myocardium both prior to and following myocardial IR.

Activation of the JAK-STAT pathway has also been demonstrated to confer cardioprotection in both *in vitro* and *in vivo* IR models and be involved in the early-phase and late-phase protection induced by IPC [21–23]. Huffman et al. [24] found that coronary effluent collected from preconditioned hearts during IPC conferred protection through activation of the JAK-STAT pathway in an isolated rat heart model. However, in another study by Breivik et al. [20] using the same isolated rat heart model, activation of the JAK-STAT pathway by preconditioned coronary effluent was not observed. Additionally, increased phosphorylation of STAT3 in the PP-IR group was also not observed in the present study, suggesting that the involvement of JAK-STAT pathway activation in the transferred myocardial protection of the preconditioned effluent or plasma collected at both early and late phase remains in question. Because the window of STAT3 activation was unclear and the timing of peak STAT3 phosphorylation remained unknown, the question of whether STAT3 phosphorylation increased during other time points requires further study.

Negoro et al. [25] found that STAT3 was activated in the myocardium after infarction and that activation of STAT3 was not confined to the ischemic infarcted area but rather was more prominently activated in the healthy border area. Based on this finding, we postulated that the survival kinase activation in the border area that was adjacent to the nonischemia area might differ from that in the ischemic central area. In the current study, we carefully divided the ischemic area into border and central areas after Evans blue staining. This showed that the preconditioned plasma increased the phosphorylation of Akt and Erk1/2 but did not increase the phosphorylation of STAT3 in both areas. However, as we performed Western blotting to detect the survival kinase of the border area myocardia using a different membrane from that containing the central area myocardia, we were unable to directly compare the phosphorylation of survival kinases between the two areas of myocardia. Additionally, there were several other limitations of this study. The rats in part 1 and part 2 were randomized separately, whereas the study would have been more ideal if they had been randomized together. Furthermore, although an *in vivo* IR model could reflect the comprehensive effects of systemic organs in the whole body better than an isolated heart model, it was difficult to utilize inhibitors of the RISK pathway in this study because of the *in vivo* model and the long time span from plasma transfusion to myocardial ischemia (plasma transfusion at 24 hours prior to ischemia). This represented a major limitation of this study.

In conclusion, this study demonstrated that the preconditioned plasma collected at the late protective phase of RIPC could activate the RISK pathway but could not activate the SAFE pathway, both prior to myocardial ischemia and following myocardial reperfusion, and both in the central area and border area of the area at risk in the myocardium. These findings suggested that the RISK pathway rather than the SAFE pathway might be involved in transferring the cardioprotection of transfused preconditioned plasma collected at the late-protective phase of RIPC. This may narrow the range of potential humoral protective factors released at the late protective phase of RIPC and facilitate their identification.

## Figures and Tables

**Figure 2:**
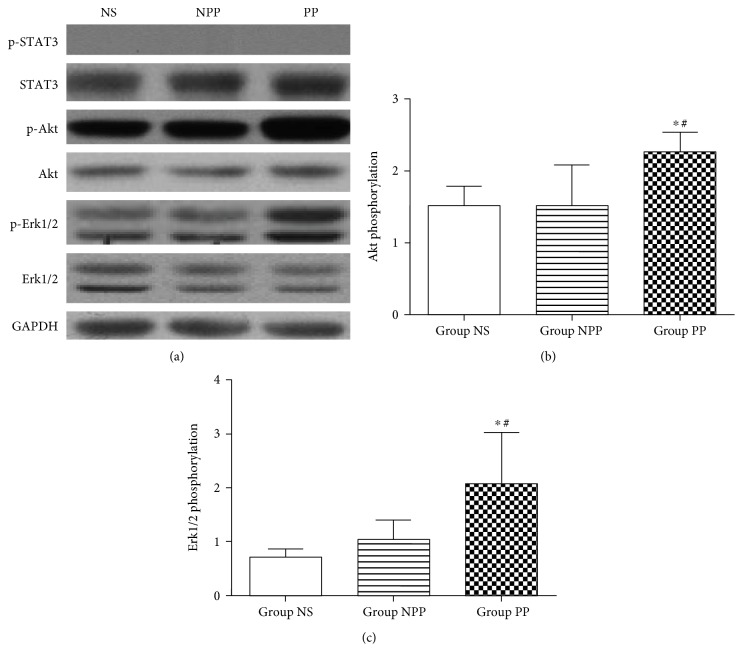
Phosphorylation of STAT3, Akt, and Erk1/2 in normal myocardium obtained after fluid transfusion but prior to myocardial ischemia reperfusion. Normal myocardium of recipient rats was collected at 24 hours after fluid transfusion. Equal amounts of protein lysate from the normal myocardium of recipient rats receiving nonpreconditioned plasma transfusion (group NPP), normal saline (group NS), or preconditioned plasma (group PP) were subjected to Western blot (*n* = 6 per group). Error bars represent the means ± SD. (a) Representative images of Western blots. Phosphorylated STAT3 (p-STAT3) and STAT3, phosphorylated Akt (p-Akt) and Akt, phosphorylated Erk1/2 (p-Erk1/2) and Erk1/2, and GAPDH are shown. Western blot bands of p-STAT3 were not observed. GAPDH was used as a loading control. (b) Phosphorylation levels of Akt, expressed as the ratio of p-Akt to Akt. (c) Phosphorylation level of Erk1/2, expressed as the ratio of p-Erk1/2 to Erk1/2. ^∗^*P* < 0.05 versus group NPP; ^#^*P* < 0.05 versus group NS.

**Figure 3:**
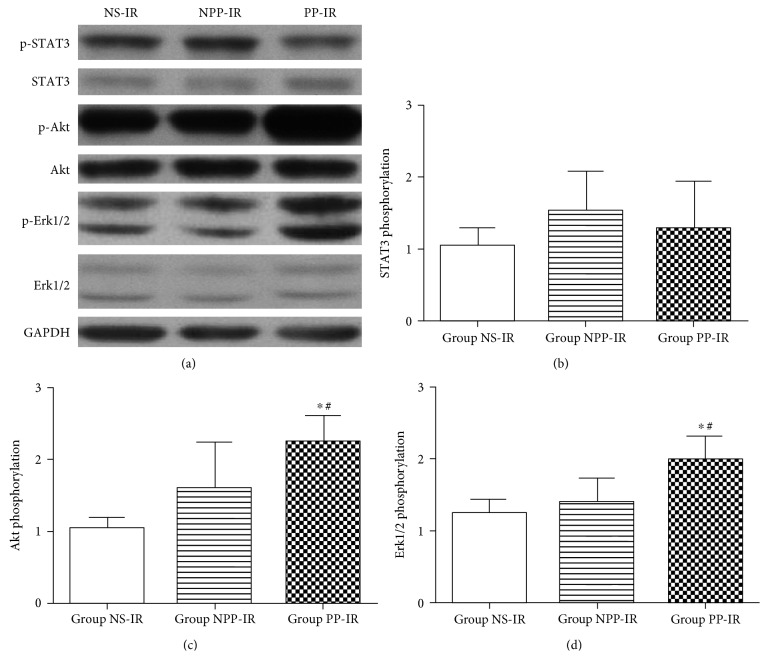
Phosphorylation of STAT3, Akt, and Erk1/2 in ischemic myocardium obtained from the central area of the area at risk. The central area of the area at risk was collected at 15 minutes after myocardial reperfusion. Equal amounts of myocardial protein lysate from the central area of the area at risk of rats receiving nonpreconditioned plasma transfusion (group NPP-IR), normal saline (group NS-IR), or preconditioned plasma (group PP-IR) were subjected to Western blot (*n* = 6 per group). Error bars represent the means ± SD. (a) Representative images of Western blots. Phosphorylated STAT3 (p-STAT3) and STAT3, phosphorylated Akt (p-Akt) and Akt, phosphorylated Erk1/2 (p-Erk1/2) and Erk1/2, and GAPDH are shown. GAPDH was used as a loading control. (b) Phosphorylation level of STAT3, expressed as the ratio of p-STAT3 to STAT3. (c) Phosphorylation level of Akt, expressed as the ratio of p-Akt to Akt. (d) Phosphorylation level of Erk1/2, expressed as the ratio of p-Erk1/2 to Erk1/2. ^∗^*P* < 0.05 versus group NPP-IR; ^#^*P* < 0.05 versus group NS-IR.

**Figure 4:**
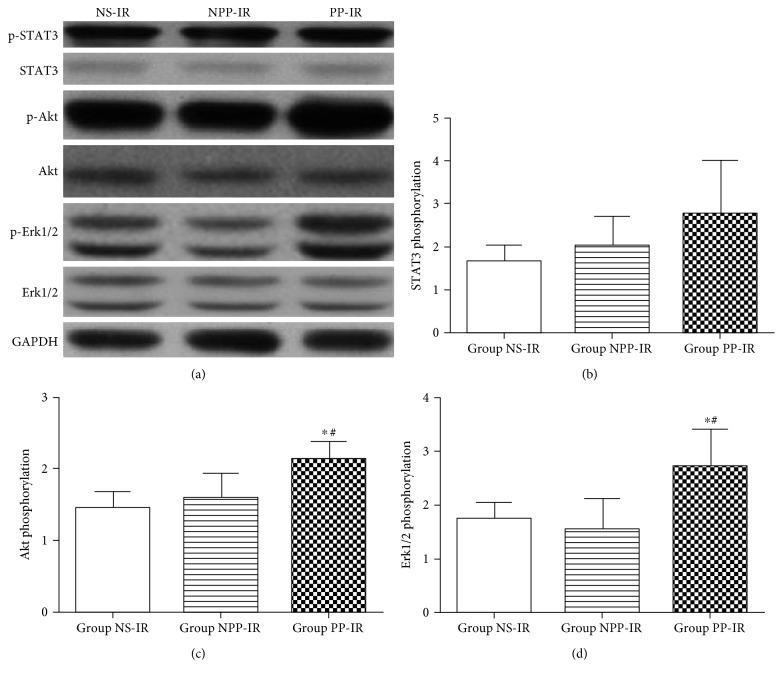
Phosphorylation of STAT3, Akt, and Erk1/2 in ischemic myocardium obtained from the border area of the area at risk. The border area of the area at risk was collected at 15 minutes after myocardial reperfusion. Equal amounts of myocardial protein lysate from the border area of the area at risk of rats receiving nonpreconditioned plasma transfusion (group NPP-IR), normal saline (group NS-IR), or preconditioned plasma (group PP-IR) were subjected to Western blot (*n* = 6 per group). Error bars represent the means ± SD. (a) Representative images of Western blots. Phosphorylated STAT3 (p-STAT3) and STAT3, phosphorylated Akt (p-Akt) and Akt, phosphorylated Erk1/2 (p-Erk1/2) and Erk1/2, and GAPDH are shown. GAPDH was used as a loading control. (b) Phosphorylation level of STAT3, expressed as the ratio of p-STAT3 to STAT3. (c) Phosphorylation level of Akt, expressed as the ratio of p-Akt to Akt. (d) Phosphorylation level of Erk1/2, expressed as the ratio of p-Erk1/2 to Erk1/2. ^∗^*P* < 0.05 versus group NPP-IR; ^#^*P* < 0.05 versus group NS-IR.
